# Supplementary material for: Spatial signatures of anesthesia-induced burst-suppression differ between primates and rodents
Source: eLife. 2022 May 24;11:e74813. doi: 10.7554/eLife.74813 (PMC9129882; doi:10.7554/eLife.74813)
Supplement: Transparent reporting form [file elife-74813-transrepform1.docx]

***eLife’s* transparent reporting form**

We encourage authors to provide detailed information *within their submission* to facilitate the interpretation and replication of experiments. Authors can upload supporting documentation to indicate the use of appropriate reporting guidelines for health-related research (see [EQUATOR Network](http://www.equator-network.org/%20)), life science research (see the [BioSharing Information Resource](https://biosharing.org/)), or the [ARRIVE guidelines](http://www.plosbiology.org/article/info:doi/10.1371/journal.pbio.1000412) for reporting work involving animal research. Where applicable, authors should refer to any relevant reporting standards documents in this form.

If you have any questions, please consult our Journal Policies and/or contact us: [editorial@elifesciences.org](mailto:editorial@elifesciences.org).

**Sample-size estimation**

- You should state whether an appropriate sample size was computed when the study was being designed
- You should state the statistical method of sample size computation and any required assumptions
- If no explicit power analysis was used, you should describe how you decided what sample (replicate) size (number) to use

Please outline where this information can be found within the submission (e.g., sections or figure legends), or explain why this information doesn’t apply to your submission:

No sample size calculations were performed. The Human and Rat 2 datasets were already acquired for previously published studies (Ranft et al. 2016 and Paasonen et al. 2016, respectively). The Macaque, Marmoset, and Rat 1 datasets were acquired at the German Primate Center as part of other ongoing studies. The fMRI data were retroactively analyzed for this study. Our goal was to exploit already available animal fMRI data.

**Replicates**

- You should report how often each experiment was performed
- You should include a definition of biological versus technical replication
- The data obtained should be provided and sufficient information should be provided to indicate the number of independent biological and/or technical replicates
- If you encountered any outliers, you should describe how these were handled
- Criteria for exclusion/inclusion of data should be clearly stated
- High-throughput sequence data should be uploaded before submission, with a private link for reviewers provided (these are available from both GEO and ArrayExpress)

Please outline where this information can be found within the submission (e.g., sections or figure legends), or explain why this information doesn’t apply to your submission:

In some subjects more than one fMRI runs were acquired. The number of fMRI runs per subject is given throughout the manuscript. We refer to the number of subjects with ‘N’, and to the number of fMRI runs with ‘n’. In the case of the Macaque and Marmoset datasets, we excluded fMRI runs with duration less than 10 minutes as well as fMRI runs in which image quality was severely compromised by imaging artifacts or motion. The decisions for data exclusion were made prior to the analysis.

**Statistical reporting**

- Statistical analysis methods should be described and justified
- Raw data should be presented in figures whenever informative to do so (typically when N per group is less than 10)
- For each experiment, you should identify the statistical tests used, exact values of N, definitions of center, methods of multiple test correction, and dispersion and precision measures (e.g., mean, median, SD, SEM, confidence intervals; and, for the major substantive results, a measure of effect size (e.g., Pearson's r, Cohen's d)
- Report exact p-values wherever possible alongside the summary statistics and 95% confidence intervals. These should be reported for all key questions and not only when the p-value is less than 0.05.

Please outline where this information can be found within the submission (e.g., sections or figure legends), or explain why this information doesn’t apply to your submission:

This information is provided in the Materials and Methods section. Additionally, figures and figure legends provide information pertaining to the displayed data.

(For large datasets, or papers with a very large number of statistical tests, you may upload a single table file with tests, Ns, etc., with reference to sections in the manuscript.)

**Group allocation**

- Indicate how samples were allocated into experimental groups (in the case of clinical studies, please specify allocation to treatment method); if randomization was used, please also state if restricted randomization was applied
- Indicate if masking was used during group allocation, data collection and/or data analysis

Please outline where this information can be found within the submission (e.g., sections or figure legends), or explain why this information doesn’t apply to your submission:

Not relevant for this study.

**Additional data files (“source data”)**

- We encourage you to upload relevant additional data files, such as numerical data that are represented as a graph in a figure, or as a summary table
- Where provided, these should be in the most useful format, and they can be uploaded as “Source data” files linked to a main figure or table
- Include model definition files including the full list of parameters used
- Include code used for data analysis (e.g., R, MatLab)
- Avoid stating that data files are “available upon request”

Please indicate the figures or tables for which source data files have been provided:

Source Data files have been provided for Figures 1G–H, Figure 2A, Figure 3B, Figure 3—figure supplement 4B, Figure 4B, Figure 4—figure supplement 5B, Figure 5B, Figure 6, and Figure 6—figure supplement 1. The custom python code implementing the carpet plots and PCA analysis is publicly available through GitHub (https://github.com/niksirbi/pcarpet/) and Zenodo (doi: 10.5281/zenodo.5545696).
